# Supplementary material for: Persistent Ethnicity-Associated Disparity in Antitumor Effectiveness of Immune Checkpoint Inhibitors Despite Equal Access
Source: Cancer Res Commun. 2022 Aug 15;2(8):806–13. doi: 10.1158/2767-9764.CRC-21-0143 (PMC9367161; doi:10.1158/2767-9764.CRC-21-0143)
Supplement: Supplementary Data — Combined supplementary data file. [file crc-21-0143-s01.pdf]

Supplementary Data

Supplementary Table 1. Patient and treatment characteristics of ICI monotherapy cohort.

		Black	White	Hispanic
**N**		58	78	29
**Age at diagnosis**		62.8	63.2	61.2
**Age at ICI initiation**		64.3	65.4	62.3
**Sex**				
	Male	47 (81.0)	61 (78.2)	17 (58.6)
	Female	11 (19.0)	17 (21.8)	12 (41.4)
**Smoking status***				
	Yes	48 (82.8)	70 (89.7)	19 (65.5)
	Never smoker	9 (15.5)	8 (10.3)	10 (34.5)
	Smoker at diagnosis	14 (24.1)	31 (39.7)	7 (24.1)
	Unknown	1 (1.7)	0 (0)	0 (0)
**Alcohol**				
	Yes	17 (29.3)	35 (44.9)	10 (34.5)
	No	36 (62.1)	33 (42.3)	19 (65.5)
	Unknown	5 (8.6)	10 (12.8)	0 (0)
**Cancer**				
	Lung	49 (84.5)	60 (76.9)	23 (79.3)
	Adenocarcinoma	34 (69.4)	36 (60.0)	17 (73.9)
	Squamous	13 (26.5)	15 (25.0)	6 (26.0)
	Small cell	2 (4.1)	6 (10.0)	0 (0)
	NSCLC (NOS)	0 (0)	3 (5.0)	0 (0)
	Head and neck SCC	9 (18.4)	18 (23.1)	6 (20.7)
**ECOG**				
	0	7 (12.1)	12 (15.4)	6 (20.7)
	1	22 (37.9)	32 (41.0)	14 (48.3)
	2	17 (29.3)	22 (28.2)	5 (17.2)
	3	3 (5.2)	6 (7.7)	3 (10.3)
	4	1 (1.7)	0 (0)	0 (0)
	Unknown	8 (13.8)	6 (7.7)	1 (3.4)
**Number of Metastasis***				
	0	8 (13.8)	11 (14.1)	4 (13.7)
	Contralateral lung only	4 (6.9)	2 (2.6)	5 (17.2)
	1	17 (29.3)	37 (47.4)	1 (3.4)
	2	21 (36.2)	14 (17.9)	15 (51.7)
	3	4 (6.9)	11 (14.1)	3 (10.3)
	4+	4 (6.9)	3 (3.8)	1 (3.4)
	Any brain metastasis	9 (15.5)	18 (23.1)	8 (27.6)
**Prior systemic therapy (all)**				
	0	9 (15.5)	14 (17.9)	5 (17.2)
	1	28 (48.3)	42 (53.8)	16 (55.2)
	2	15 (25.9)	14 (17.9)	4 (13.8)
	3	4 (6.9)	4 (5.1)	1 (3.4)
	4+	2 (3.4)	4 (5.1)	3 (10.3)

**Prior systemic therapy (concurrent and adjuvant chemo excluded)**				
	0	12 (20.7)	21 (26.9)	6 (20.7)
	1	29 (50.0)	43 (55.1)	15 (51.7)
	2	12 (20.7)	7 (9.0)	4 (13.8)
	3	3 (5.2)	3 (3.8)	1 (3.4)
	4+	2 (3.4)	4 (5.1)	3 (10.3)
**ICI treatment**				
	Nivolumab	36 (62.1)	46 (59.0)	15 (51.7)
	Pembrolizumab	22 (37.9)	32 (41.0)	14 (48.3)
**Prior radiation anywhere***				
	Yes	39 (67.2)	63 (80.8)	17 (58.6)
	No	19 (32.8)	15 (19.2)	12 (41.4)
**PD-L1 status (NSCLC)**				
	0%	2 (4.3)	4 (7.4)	4 (17.4)
	1-49%	7 (14.9)	4 (7.4)	3 (13.0)
	>50%	12 (25.5)	14 (25.9)	7 (30.4)
	Unknown	26 (55.3)	32 (59.3)	9 (39.1)
*p<0.05 for Hispanic vs. White				

Supplementary Figure 1. Patient and disease characteristics with significant differences among the race/ethnic groups for the entire cohort (ICI monotherapy and chemo/ICI combination therapy). A) Smoking status, **B)** alcohol consumption, and **C)** number of metastatic sites.

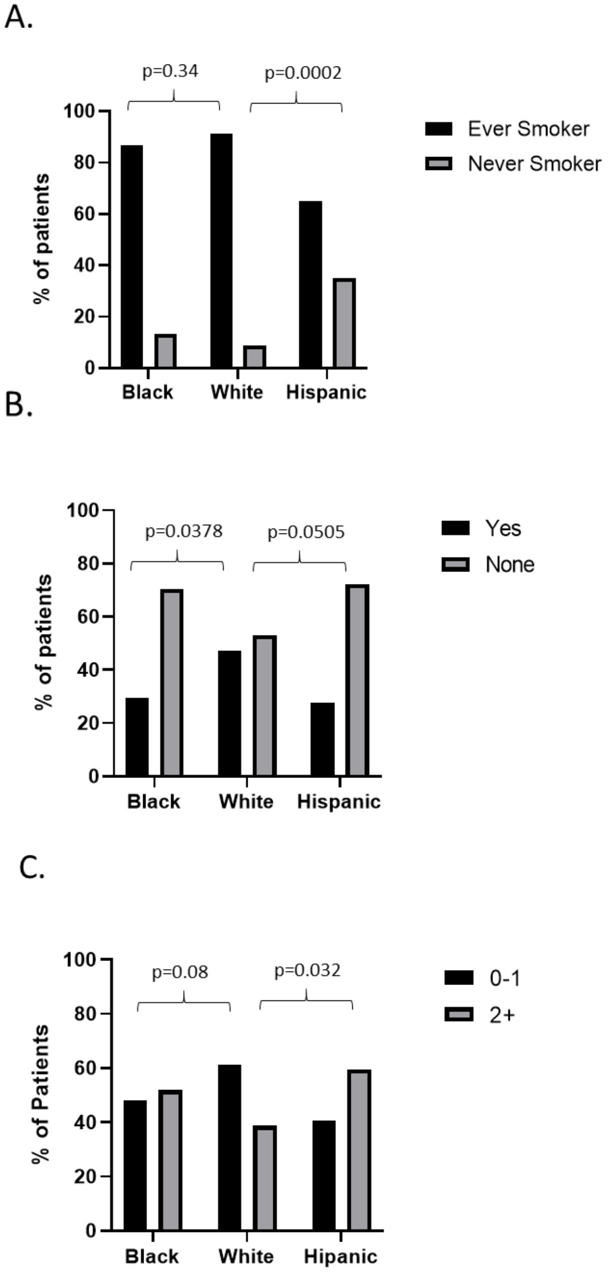

Supplementary Figure 2. Cohort characteristics with significant differences among the race/ethnic groups for the ICI monotherapy cohort. A) Smoking status and **B)** number of metastatic sites.

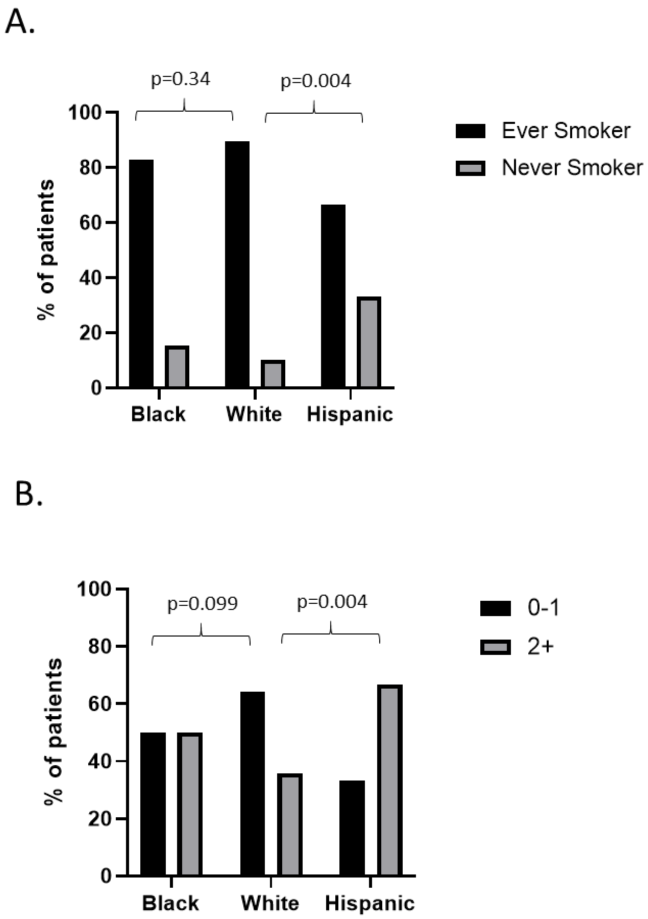

Supplementary Figure 3. Radiation treatment differed significantly as a function of race/ethnicity. A) entire cohort and **B)** ICI monotherapy cohort.

A.

B.

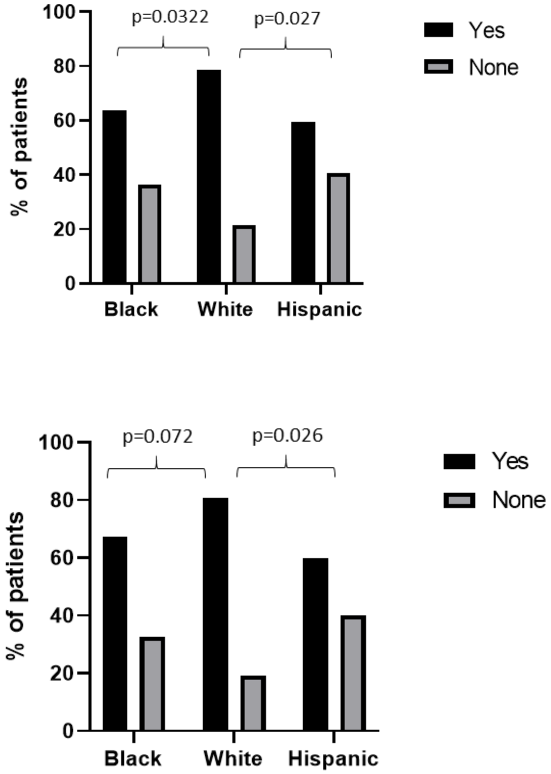

Supplementary Figure 4. ORR as a function of cohort characteristics with significant differences among the race/ethnic groups for the entire cohort (ICI monotherapy and chemo/ICI combination therapy). A) Smoking status, **B)** alcohol consumption, **C)** number of metastatic sites, and **D)** history of radiation treatment. CR- complete response; PR- partial response; SD- stable disease; PD- progressive disease.

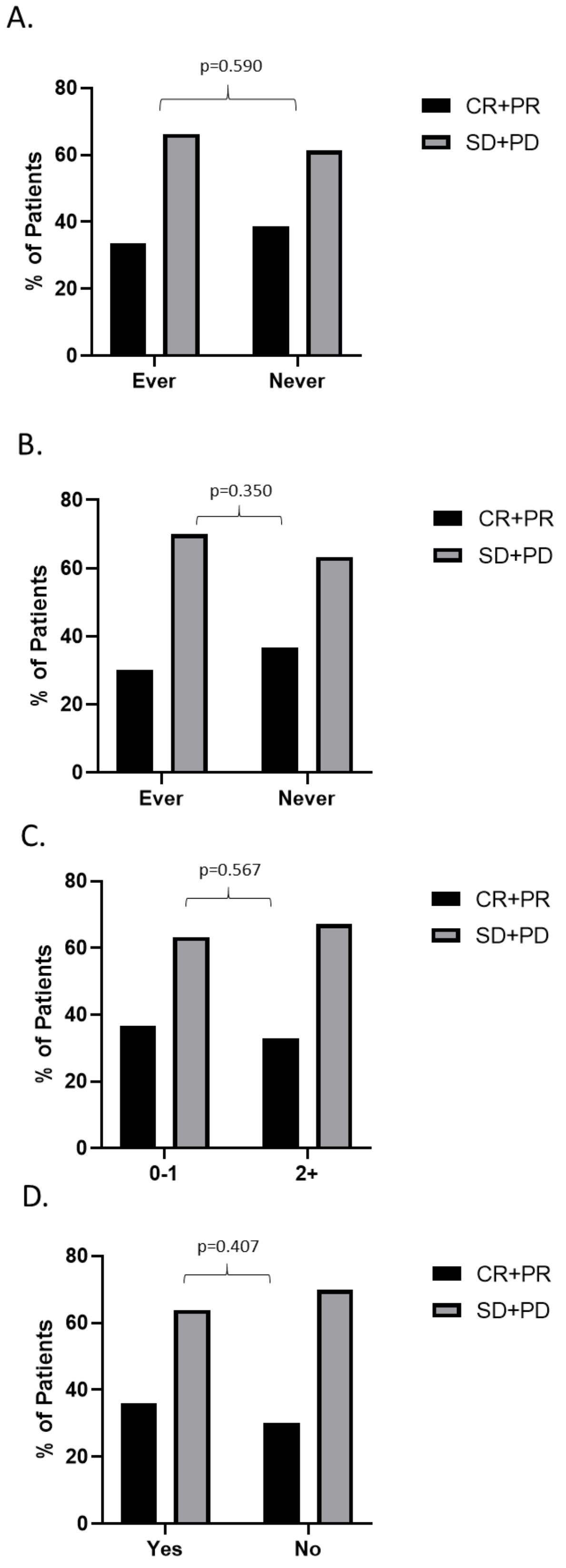

Supplementary Figure 5. ORR as a function of cohort characteristics with significant differences among the race/ethnic groups for the ICI monotherapy cohort. A) Smoking status, **B)** number of metastatic sites, and **C)** history of radiation treatment. CR- complete response; PR- partial response; SD- stable disease; PD- progressive disease.

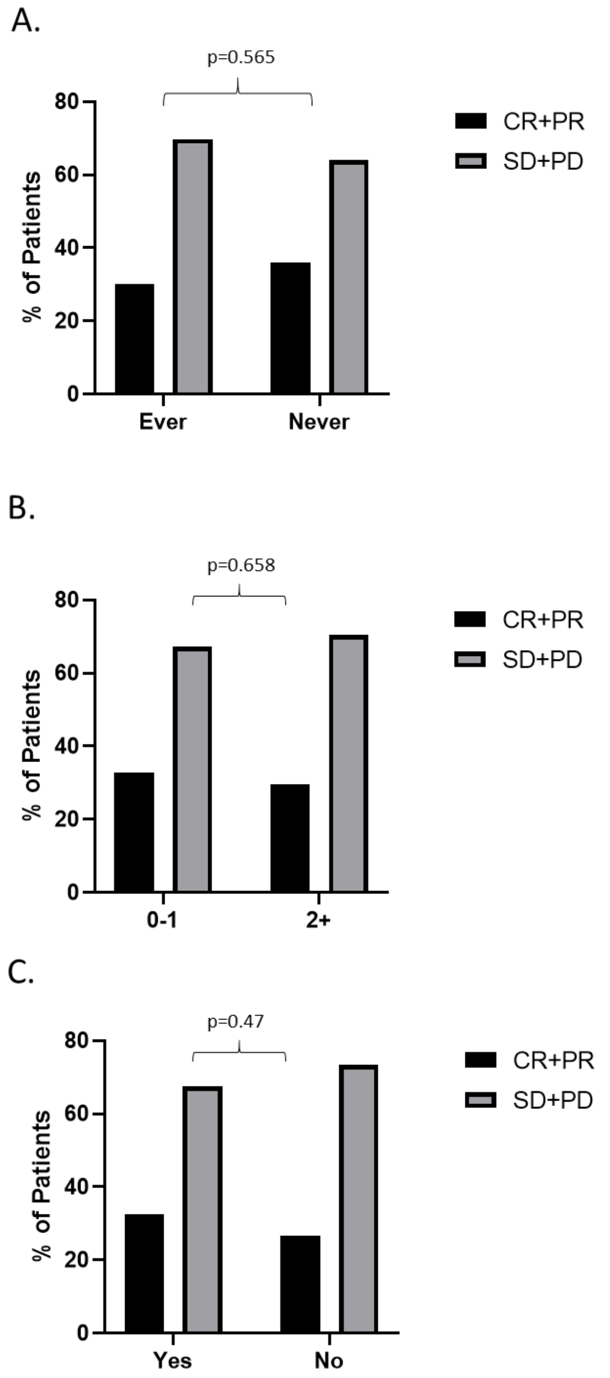

Supplemental Figure 6: Toxicity and treatment type in the entire cohort. A) treatment type by race/ethnicity and **B)** toxicity by treatment type. P values were determined by two-sided Chi-square tests.

A.

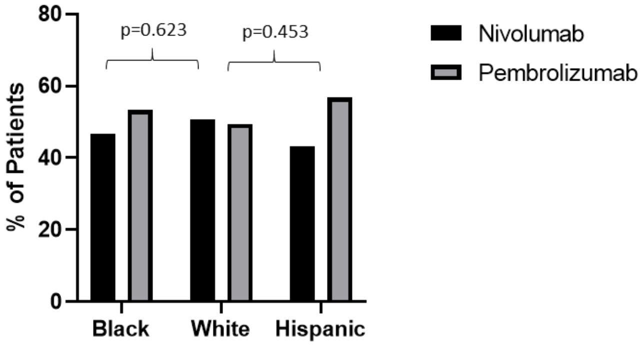

B.

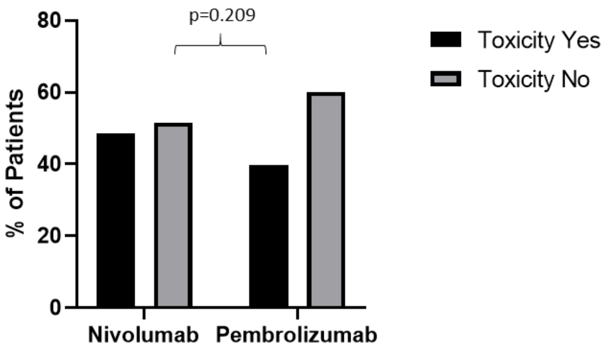

Supplemental Figure 7: Toxicity and treatment type in the monotherapy cohort. A) treatment type by race/ethnicity and **B)** toxicity by treatment type. P values were determined by two-sided Chi-square tests

A.

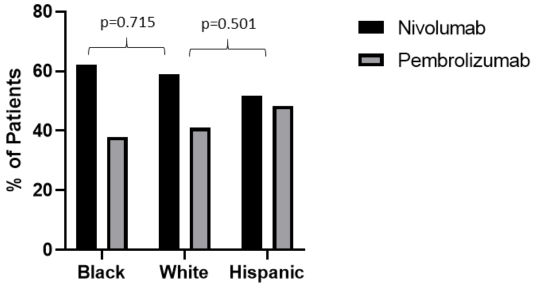

B.

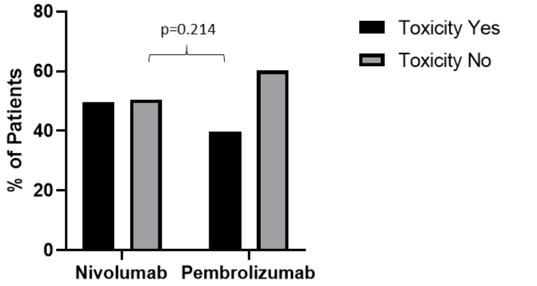

Supplementary Table 2. p16 status and response among oropharyngeal cancers by race/ethnicity.

Race/Ethnicity	p16 status	Response type
Black	unknown	Progression
Black	unknown	Progression
White	p16+	Partial response
White	p16+	Progression
White	p16+	Progression
White	p16+	Progression
White	p16-	Progression
White	p16-	Progression
Hispanic	p16+	Progression

Supplementary Table 3. Severe irAEs resulting in steroid administration or ICI cessation with summary of specific severe irAEs.

	Black	White	Hispanic
**irAE requiring stop of IO and/or systemic steroids**	15 (19.5)	16 (17.2)	6 (15.8)
Stop/hold IO	10	8	4
Steroid only	5	8	2
**irAE type**			
Ataxia	2	0	0
Meningioencephalitis	1	0	0
Hypophisitis	0	1	0
Adrenal insufficency	1	1	0
Pneumonitis	3	3	3
Tracheobronchitis	0	1	0
Colitis	3	3	2
Hepatitis	2	2	1
Renal Toxicity	0	1	0
Rash	1	2	0
Shingles	1	0	0
ITP	1	0	0
Arthritis	0	2	0
